# Assessing platelet-lymphocyte ratio in EGFR-mutated non-small cell lung cancer patients treated with tyrosine kinase inhibitors: An analysis across TKI generations

**DOI:** 10.21203/rs.3.rs-4930668/v1

**Published:** 2024-10-16

**Authors:** Ryan Cooper, Dhruv Ramaswami, Jacob Thomas, Jorge Nieva, Robert Hsu

**Affiliations:** University of Southern California Keck School of Medicine; University of Southern California; USC Norris Comprehensive Cancer Center; USC Norris Comprehensive Cancer Center; USC Norris Comprehensive Cancer Center

## Abstract

**Introduction:**

The prognostic utility of laboratory markers in patients with non-small cell lung cancer (NSCLC) harboring *EGFR* mutations treated with tyrosine kinase inhibitors (TKIs) is an ongoing area of research. The utility of the platelet-lymphocyte ratio (PLR) in patients treated with osimertinib is undetermined.

**Methods:**

151 patients treated with *EGFR* TKIs in Los Angeles were grouped into one of two groups according to generation of TKI. Differences in progression free survival (PFS) by stratification by PLR was determined using Kaplan-Meier analysis. Differences in median change in laboratory markers by generation of TKI was analyzed using Mann-Whitney tests. Cox Hazard Regression was used to perform multivariate analysis.

**Results:**

Median PFS of those managed with 1st or 2nd generation TKIs was significantly lower in patients with a PLR ≥ 180 (10.5 months) compared to those with PLR < 180 (16.6 months, p = 0.0163). Median PFS was comparable in those treated with osimertinib regardless of PLR. Patients managed with osimertinib had a significant decrease in absolute lymphocyte count (ALC) at 6 weeks and in platelets at 6 weeks and 3 months compared to those managed with 1st or 2nd generation TKIs.

**Discussion:**

The prognostic value of PLR was more apparent in patients treated with 1st or 2nd generation TKIs compared to those treated with osimertinib. Third generation *EGFR* TKIs may be more efficacious in treating patients with laboratory findings previously shown to predict poor survival. The significant changes in peripheral cell counts suggest variability tumor microenvironment changes dependent on the generation of TKI received.

## 1.0. Introduction

Lung cancer is currently the leading cause of cancer related deaths worldwide, contributing to an estimated 125,070 deaths and 234,580 new diagnosed cases projected in 2024 alone [1]. Non-small Cell Lung Cancer (NSCLC) specifically accounts for approximately 80% of all lung cancer cases [1]. Beginning in the early 2000s, patients harboring epidermal growth factor receptor (*EGFR*) mutations have multiple targeted therapy options available that have drastically improved survival outcomes in this patient population [2, 3]. For patients treated with *EGFR* tyrosine kinase inhibitors (TKIs), such as gefitinib, erlotinib, and osimertinib, techniques for predicting survival outcomes by utilizing pre-treatment clinical and laboratory data remains an active area of research. Improved foresight into each patient’s clinical course by using relatively inexpensive and easily obtainable laboratory and clinical values can help dictate treatment decisions and optimize clinical decision making.

Possible prognostic markers for patients with NSCLC include the neutrophil-lymphocyte ratio (NLR) and the platelet-lymphocyte ratio (PLR). Peripheral cell ratios have been demonstrated as significant prognostic biomarkers in a variety of cancer types [4–7] and in NSCLC patients receiving immunotherapy and systemic chemotherapy [8–13]. Platelet-lymphocyte ratio (PLR) specifically has also been investigated as a possible pre-treatment biomarker in NSCLC that may be useful for prognostic purposes [14–16].

It is well known that chronic systemic inflammation is associated with worse survival outcomes [17–19]. Elevated NLR and PLR have thus been hypothesized to serve as an easily obtainable surrogate marker for degree of systemic inflammation [20]. While multiple studies have demonstrated that higher pre-treatment NLR in patients treated with *EGFR* TKIs are significant predictors of overall and progression free survival [8, 21–25], the results are not definitive [11]. The efficacy of pre-treatment peripheral laboratory markers thus remains an active area of debate.

Osimertinib has demonstrated superior efficacy in survival outcomes in the first-line setting and in management of patients harboring the T790M resistance mutation [26]. As of 2024, osimertinib is currently approved in the adjuvant setting in patients with exon 19 deletions or L858R mutations treated with tumor resection, in the first line setting in patients harboring exon 19 deletions or L858R mutations either as monotherapy or in combination with pemetrexed or platinum-based chemotherapy, or in the second-line setting in patients who progressed on previous EGFR TKI therapies who harbor the T790M resistance mutation [27].

A study by Liu et al. reported that elevated PLR was associated with worse progression free survival (PFS) in patients treated with *EGFR* TKIs; however, patients in this study were limited to management with 1st or 2nd generation TKIs [28]. This study aims to elucidate the prognostic significance of pre-treatment laboratory ratios, namely PLR, depending on whether patients received either a 1st or 2nd generation EGFR TKI (e.g. gefitinib, afatinib, erlotinib) or a 3rd generation EGFR TKI (e.g. osimertinib) and to identify significant peripheral immune cell changes over the course of patient treatment.

## 2.0. Methods

### 2.1. Patient selection

**Figure 1:**
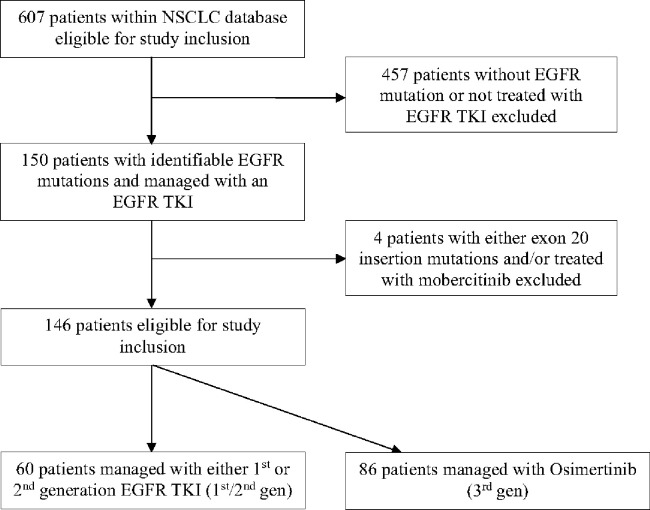
Patient selection algorithm

This study was granted approval by the University of Southern California’s Institutional Review Board. The need for informed consent was waived as data analysis was retrospective in nature. Patient information was obtained from a database consisting of 607 NSCLC patients treated at Norris Comprehensive Cancer Center, Keck Hospital of USC, or Los Angeles General Medical Center with comprehensive genomic profiling from 2015 to 2023. Those with known *EGFR* mutations identified by next-generation sequencing (NGS) and managed with an *EGFR* TKI were selected for study inclusion. Patients with exon 20 insertions were excluded from further analysis. Patients were further subdivided into one of two categories according to the generation of the first *EGFR* TKI administered: 1st /2nd generation (1st /2nd gen) or 3rd generation (3rd gen). The process for patient selection and organization are summarized in Fig. 1.

### 2.2. Data collection:

Patient demographic, clinical, and laboratory data were collected from each hospital’s electronic medical record and recorded in an electronic database. Laboratory values of interest included absolute neutrophil count (ANC), absolute lymphocyte count (ALC), platelet count, albumin, and body mass index (BMI). Each of these values were recorded at multiple different time points over the course of each patient’s EGFR TKI treatment within a four-week range, starting at the time of treatment initiation up to 12 months. The platelet-lymphocyte ratio (PLR, defined as the fraction of platelet count to ALC) was calculated at each time point. Information about each patient’s progression status and date of progression were similarly recorded in the electronic database with progression defined as noted on imaging reports or by clinical documentation from the patient’s provider.

### 2.3. Statistical analysis:

All statistical analyses were performed using GraphPad Prism Version 10.1.1. Both the 1st /2nd gen and 3rd gen groups were stratified by either PLR < 180 or PLR ≥ 180, using the study by MacDonald et al. as reference[14]. We calculated and reported differences in median progression free survival (PFS). Differences in progression free survival (PFS) for each treatment group were plotted on Kaplan-Meier curves and analyzed using Log-Rank tests. Each sub-group was further stratified by BMI, using a cut-off of 21, and albumin, using a cut-off of 3.2, and subsequently analyzed for differences in progression free survival using Log-Rank tests. Multivariate analysis was performed using Cox-proportional hazard regression, specifically assessing for the impact of the following demographic and clinical characteristics on progression free survival: sex, ethnicity, smoking status, primary tumor histology, presence of brain metastases, BMI, age at start of treatment, Albumin < 3.2 or ≥ 3.2, and PLR < 180 or ≥ 180. Differences in PLR, ANC, ALC, and platelet count were calculated for each patient at 6 weeks, 3 months, 6 months, and 12 months compared to each laboratory value at the time of treatment initiation. Significant differences in the median change of each of these laboratory values, between those who received 1st /2nd gen or 3rd gen *EGFR* TKIs, were assessed using Mann-Whitney tests.

## 3.0. Results

### 3.1. Patient Summary:

Patient demographic and clinical data are outlined in Table 1. The median age at the initiation of TKI treatment for the 1st /2nd generation group was 62.4 years compared to 67.1 years for the 3rd gen group. Most patients in the 1st /2nd gen group received erlotinib (n = 40) followed by afatinib (n = 18) and gefitinib (n = 2). All patients in the 3rd generation group received osimertinib as their first-line EGFR TKI (n = 86). The most common EGFR mutation in both groups was an exon 19 deletion (n = 31 and 45 for the 1st /2nd gen and 3rd gen groups respectively), followed by the L858R mutation (n = 15 and 33). Fewer patients had less common EGFR mutations, defined as “uncommon” mutations, or two distinct identifiable EGFR mutations, defined as “compound” mutations. Most patients in both groups were metastatic at TKI initiation (n = 57 and 77 in the 1st /2nd gen and 3rd gen groups respectively) and received EGFR TKIs in the first line setting (n = 51 and 76).

### 3.2. Prognostic impact of platelet-lymphocyte ratio:

**Figure 2:**
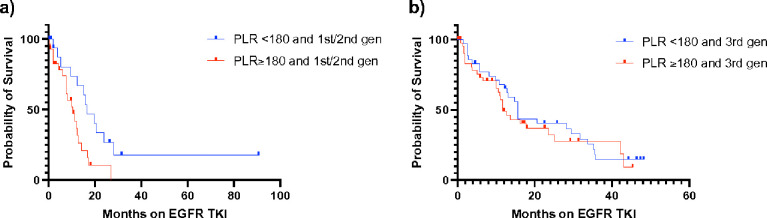
Kaplan-Meier survival curve of PFS separated by generation of EGFR TKI received and platelet-lymphocyte ratio. ***a*)** Median PFS of 1^st^/2^nd^ gen, PLR <180 = 16.6 months vs. median PFS of 1^st^/2^nd^ gen, PLR ≥180 = 10.5 months (p = 0.0163). ***b*)** Median PFS of 3^rd^ gen, PLR <180 = 15.7 months vs. median PFS of 3^rd^ gen, PLR ≥180 = 11.7 months (p = 0.4932)

Patients in the 1st /2nd gen group with a higher PLR had a significantly lower median PFS (10.5 months) compared to those with a lower PLR (16.6 months; p = 0.0163). The difference in PFS was not as readily apparent in the 3rd gen group: median PFS of 11.7 months and 15.7 months in the higher and lower PLR groups respectively (p = 0.4932) (Fig. 2).

### 3.3. PLR stratified by BMI and Albumin:

We sought to determine whether further stratification of the data outlined above by BMI and albumin would further predict PFS (**Online Resource 1)**. The total number of patients within each subgroup is presented in **Online Resource 2**.

For patients in the 1st /2nd gen group with PLR ≥ 180 and BMI < 21, median PFS was 12 months compared to 15.9 months in those with both PLR < 180 and BMI ≥ 21 (p = 0.2630). Similarly, the same analysis performed on the 3rd gen group yielded similar results: median PFS of 12.2 months in the PLR ≥ 180 and BMI < 21 group versus median PFS of 15.7 months in the PLR < 180 and BMI ≥ 21 group (p = 0.1202). In the 1st /2nd gen group, those with both PLR ≥ 180 and albumin < 3.2 had a median PFS of 9.4 months compared to 16.6 months in those with PLR < 180 and albumin ≥ 3.2 (p = 0.2863). Evaluation of the median PFS of those in the 3rd gen group was limited due to low sample size.

### 3.4. Differences in peripheral cell counts by generation of EGFR TKI

To explain the differences in prognostic efficacy of the laboratory markers identified above, we determined whether the overall difference in peripheral cell counts and cell ratios at different time points varied depending on the generation of *EGFR* TKI received (Table 2). The differences in both NLR and PLR at 6 weeks, 3 months, 6 months, and 12 months after treatment initiation were not significant. However, when evaluating the median differences in ANC (ΔANC), ALC (ΔALC), and platelet count (Δplatelet) at each of these time points, the median change of each of these three lab values in the 3rd gen group appeared to be greater when compared to the 1st /2nd gen group, with all three values showing a greater decline over time in the 3rd gen group. Specifically, results were statistically significant (α = 0.05) when evaluating change in ANC at 3 months (ΔANC = −0.51 in 1st /2nd gen and − 1.67 in 3rd gen; p = 0.0259), ALC at 6 weeks (ΔALC = −0.07 in 1st /2nd gen and − 0.20 in 3rd gen; p = 0.0480), and platelets at 6 weeks (Δplatelet = −26.0 in 1st /2nd gen and − 60.0 in 3rd gen; p = 0.0042) and 3 months ( Δplatelet = −14.5 in 1st /2nd gen and − 55.0 in 3rd gen; p = 0.0095). While the differences in these values were not statistically significant at the other time points, results approached significance with similar trends.

### 3.5. Multivariate analysis of laboratory and clinical markers on PFS

Multivariate analysis is presented in Table 3. Controlling for confounding variables, smoking status (HR 15.12, 95% CI 2.467 to 118.1) and presence of brain metastases (HR 5.940, 95% CI 2.082 to 18.00) were both associated with worse progression free survival in patients treated with 1st /2nd generation *EGFR* TKIs. In contrast, only male sex (HR 2.515, 95% CI 1.147 to 5.513) was associated with worse progression free survival in patients treated with 3rd gen *EGFR* TKIs.

## 4.0. Discussion

Though our results indicate that elevated PLR was significantly predictive of worse PFS in patients treated with 1st or 2nd generation *EGFR* TKIs, comparable to the study conducted by Liu et al.[28], the relationship was not apparent in patients managed with 3rd gen *EGFR* TKIs. Our study suggests that osimertinib may be equally efficacious in patients regardless of negative prognostic markers. Though clinical data supports the superior survival outcomes of osimertinib compared to 1st /2nd generation TKIs [26], an explanation for the discrepancy in laboratory markers is not currently identified in the literature.

Prior studies have identified changes in the tumor microenvironment in response to management with *EGFR* TKIs, resulting in changes including alterations in the populations of tumor infiltrating lymphocytes (TILs), such as an increase number of effector CD8 + T cells and increased antitumor CD4 + T cells, increased M1 tumor-associated macrophages (TAMs) and decreased M2 TAMs, increased MHC I and MHC II expression, and increased secretion of pro-inflammatory cytokines [29, 30]. Additionally, it has been previously found that improvements in survival outcomes in multiple cancer types correlate with changes in peripheral cell counts, specifically an overall decrease in platelet count and an increase in ALC [5, 9, 10, 12, 31–33]. However, differences in tumor microenvironment changes dependent on the generation of *EGFR* TKI are not currently defined in the literature.

Our findings revealing changes in laboratory values at different time points in patients treated with 3rd gen TKIs compared to patients treated with 1st /2nd gen TKIs may suggest differences in tumor microenvironment response and the systemic pro-inflammatory state depending on the generation of TKI received. Though these reported changes were not statistically significant, they reveal an interesting trend. While the decrease in ANC and platelet count in patients managed with osimertinib are consistent with the laboratory changes associated with improved survival outcomes, the decrease in ALC, most notably at 6 weeks after treatment initiation, is surprising since prior studies have identified an increase in ALC correlating with improved survival and that a decrease in ALC over time could be suggestive of decreased T cell infiltration and activation as seen in osimertinib resistance [34]. This suggests that the efficacy of *EGFR* TKIs may be dependent on a functional immune system. However, because lymphocytes are inherently heterogenous, the reliability of using ALC as a biomarker for improved survival may have limited utility.

Further stratification of PLR by additional clinical variables, particularly BMI, trended towards worse median progression free survival identifiable in the 1st /2nd gen group. Though results were not statistically significant, we believe combining multiple clinical prognostic markers may warrant further investigation using larger sample sizes.

Notably, our multivariate analysis failed to identify a single laboratory biomarker associated with worse PFS in either treatment group while holding all other variables constant. This suggests that the reliance on a single laboratory datapoint to accurately prognosticate patients treated with *EGFR* TKIs may be limited, particularly in patients managed with osimertinib. We therefore suggest PLR be used in combination with other variables, such as performance status, to accurately predict pre-treatment survival outcomes.

Our study has notable limitations, particularly due to its retrospective study design. Additionally, our conclusions resulting from stratifying PLR by albumin and BMI are limited by low sample size. Last, patients who were treated with osimertinib were generally treated in more recent years compared to patients treated with 1st /2nd gen *EGFR* TKIs due to its FDA approval in 2018 for use in *EGFR* mutated patients in the first-line setting.

Further studies evaluating the tumor microenvironment differences in patients receiving 3rd generation *EGFR* TKIs versus 1st /2nd generation *EGFR* TKIs are warranted to help illustrate how 3rd gen TKIs may be more efficacious. Additionally, more studies may help determine if PLR should be considered as a reliable outcome predictor in *EGFR* mutant NSCLC patients receiving TKIs and to better understand trends in peripheral cell counts in patients treated with 3rd generation *EGFR* TKIs.

## 5.0. Conclusion

Pre-treatment PLR may be useful to determine prognosis in patients treated with 1st or 2nd generation *EGFR* TKIs. A combination of multiple clinical variables, such as PLR and BMI, may be effective in predicting clinical response in patients treated with osimertinib, though further studies with greater statistical power may be necessary. Alterations in peripheral cell counts over the duration of treatment with *EGFR* TKIs, including decreased ANC, ALC, and platelets, may be more readily apparent in patients treated with 3rd gen TKIs compared to those treated with 1st or 2nd gen TKIs, possibly suggesting differences in tumor microenvironment response.

## Figures and Tables

**Table 1:** Summary of demographic and clinical data of patients treated depending on generation of TKI received

	1st /2nd Generation TKI (n = 60)	3rd Generation TKI (n = 90)

**Age at diagnosis (median, SD)**	61.5 (13.2)	66 (11.7)

**Age at TKI initiation (median, SD)**	62.4 (20.3)	67.1 (12.3)

**Sex (n)**	*Male*: 21	*Male*: 28
	*Female*: 39	*Female*: 58

**Ethnicity (n)**	*Hispanic*: 17	*Hispanic*: 14
	*Asian/Pacific Islander*: 28	*Asian/Pacific Islander*: 45
	*Black*: 1	*Black*: 4
	*White*: 13	*White*: 18
	*Unknown/other*: 1	*Unknown/other*: 5

**Smoking Status (n)**	*Never smoker*: 47	*Never smoker*: 64
	*Ever smoker*: 13	*Ever smoker*: 22

**Tumor Histology (n)**	*Adenocarcinoma*: 56	*Adenocarcinoma*: 80
	*Squamous*: 1	*Squamous*: 2
	*Adenosquamous*: 2	*Adenosquamous*: 3
	*NOS*: 1	*NOS*: 1

**Initial EGFR TKI (n)**	*Erlotinib*: 40	*Osimertinib*: 86
	*Afatinib*: 18	
	*Gefitinib*: 2	

**EGFR Mutation/s (n)**	*Exon 19 ins/del*: 31	*Exon 19 ins/del*: 45
	*L858R*: 15	*L858R*: 33
	*“Rare” Mutation*: 8	*“Rare” Mutation*: 4
	*“Compound” Mutation*: 2	*“Compound” Mutation*: 4
	*Unknown/Data not accessible*: 4	*Unknown/Data not accessible*: 0

**Cancer stage at TKI initiation (n)**	*Stage 1*: 0	*Stage 1*: 3
	*Stage 2*: 1	*Stage 2*: 4
	*Stage 3*: 2	*Stage 3*: 2
	*Stage 4*: 57	*Stage 4*: 77

**Brain mets at TKI initiation? (n)**	*No brain mets*: 36	*No brain mets*: 59
	*+brain mets*: 24	*+brain mets*: 27

**Line of Therapy (n)**	*1st line*: 51	*1st line*: *76*
	*2nd line or greater*: 9	*2nd line or greater*: 10

**BMI at TKI initiation (n)**	*<21*: 15	*<21*: 16
	≥21: 36	*≥21*: 67
	Data unavailable: 9	*Data unavailable*: 3

**NLR at TKI initiation (n)**	*NLR < 5*: 29	*NLR < 5*: 43
	*NLR ≥ 5*: 28	*NLR ≥ 5*: 33
	*Data unavailable*: 13	*Data unavailable*: 10

**PLR at TKI initiation (n)**	*PLR < 180*: 18	*PLR < 180*: 34
	*PLR ≥ 180*: 29	*PLR ≥ 180*: 42
	*Data unavailable*: 13	*Data unavailable*: 10

**Albumin at TKI initiation (n)**	*Albumin < 3.2*: 6	*Albumin < 3.2*: 6
	*Albumin ≥ 3.2*: 40	*Albumin ≥ 3.2*: 64
	*Data unavailable*: 14	*Data unavailable*: 16

**Table 2:** Median change in laboratory parameters depending on generation of EGFR TKI administered. Statistical significance assessed with Mann-Whitney tests (α = 0.05)

	1st /2nd Gen	3rd Gen	P -value

Median ΔNLR (n):	−0.80 (35)	−0.80 (60)	0.934
At 6 weeks	−0.20 (38)	−1.00 (62)	0.701
At 3 months	−0.85 (26)	−0.90 (51)	0.934
At 6 months	−0.55 (16)	−0.70 (38)	0.870
At 12 months			

Median ΔPLR (n):	−16.40 (35)	−5.05 (60)	0.803
At 6 weeks	−0.75 (38)	−11.30 (62)	0.931
At 3 months	−25.05 (26)	−24.60 (51)	0.526
At 6 months	−23.80 (16)	−25.20 (38)	0.754
At 12 months			

Median ΔANC (n):	−1.21 (35)	−1.54 (60)	0.144
At 6 weeks	−0.51 (38)	−1.67 (62)	0.026*
At 3 months	−1.48 (26)	−2.61 (51)	0.093
At 6 months	−0.65 (16)	−1.22 (38)	0.061
At 12 months			

Median ΔALC (n):	−0.07 (35)	−0.20 (60)	0.048*
At 6 weeks	0.00 (38)	−0.22 (62)	0.058
At 3 months	0.10 (26)	−0.16 (51)	0.077
At 6 months	0.03 (16)	−0.26 (38)	0.191
At 12 months			

Median ΔPlatelets (n):	−26.0 (35)	−60.0 (61)	0.004**
At 6 weeks	−14.5 (38)	−55.0 (64)	0.010**
At 3 months	−49.0 (27)	−75.0 (55)	0.274
At 6 months	−31.0 (16)	−55.0 (39)	0.252
At 12 months			

**Table 3:** Multivariate analysis using cox-proportional hazard model to analyze impact of demographic and clinical parameters on progression-free survival according to generation of EGFR TKI received

Variable	1st /2nd Gen, HR	1st /2nd Gen, 95% CI	3rd Gen, HR	3rd Gen, 95% CI

Sex	Reference	0.068 to 1.553	Reference	1.147 to 5.513
Female	0.3762		2.515	
Male				

Ethnicity	Reference	0.840 to 7.863	Reference	0.566 to 2.998
Asian/Pacific Islander	2.462		1.315	
Other				

Smoking Status	Reference	2.467 to 118.1	Reference	0.435 to 2.173
Never smoker	15.12		1.006	
Ever smoker				

Tumor Histology	Reference	0.248 to 22.64	Reference	0.875 to 15.90
Adenocarcinoma	2.752		4.271	
Other				

Brain Mets	Reference	2.082 to 18.00	Reference	0.652 to 2.789
No	5.940		1.379	
Yes				

BMI	0.896	0.796 to 1.00	0.969	0.895 to 1.039

Age at Treatment Initiation	0.981	0.943 to 1.019	1.024	0.996 to 1.055

Albumin	Reference	0.115 to 2.938	Reference	0.139 to 4.093
≥ 3.2	0.673		0.971	
< 3.2				

NLR	Reference	0.198 to 2.021	Reference	0.309 to 1.255
< 5	0.653		0.631	
≥ 5				

PLR	Reference	0.521 to 6.010	Reference	0.634 to 3.981
< 180	1.789		1.598	
≥ 180				

## Data Availability

The data that support the findings of the study are not openly available to support anonymity of our patients included in analysis. Deidentified data is available from the corresponding author upon reasonable request. Data is stored in a secured online database at the University of Southern California.
